# An Overview of Canine Inherited Neurological Disorders with Known Causal Variants

**DOI:** 10.3390/ani13223568

**Published:** 2023-11-18

**Authors:** Vlad Cocostîrc, Anamaria Ioana Paștiu, Dana Liana Pusta

**Affiliations:** Department of Genetics and Hereditary Diseases, Faculty of Veterinary Medicine, University of Agricultural Sciences and Veterinary Medicine Cluj-Napoca, 400372 Cluj-Napoca, Romania; oana.pastiu@usamvcluj.ro (A.I.P.); dana.pusta@usamvcluj.ro (D.L.P.)

**Keywords:** canine inherited neurological diseases, causal variant, Mendelian disorders

## Abstract

**Simple Summary:**

This review explores various inherited neurological conditions found in dogs, which can be present from birth or develop later in life. Researchers use two main methods to identify the genetic causes of these diseases: wide-ranging genetic investigations and focused investigations on specific genes. Online Mendelian Inheritance in Animals currently lists 418 Mendelian disorders in dogs, and 355 of them have their likely genetic causes identified. Most of the reviewed neurological disorders follow an autosomal recessive inheritance pattern, which means that both parents must carry the faulty gene for their offspring to develop the disease. Some dog breeds are more susceptible to these conditions, with the Golden Retriever and Belgian Shepherd having multiple known inherited neurological disorders. The use of DNA tests is crucial in managing and eventually eliminating these inherited diseases in dogs.

**Abstract:**

Hereditary neurological conditions documented in dogs encompass congenital, neonatal, and late-onset disorders, along with both progressive and non-progressive forms. In order to identify the causal variant of a disease, the main two approaches are genome-wide investigations and candidate gene investigation. Online Mendelian Inheritance in Animals currently lists 418 Mendelian disorders specific to dogs, of which 355 have their likely causal genetic variant identified. This review aims to summarize the current knowledge on the canine nervous system phenes and their genetic causal variant. It has been noted that the majority of these diseases have an autosomal recessive pattern of inheritance. Additionally, the dog breeds that are more prone to develop such diseases are the Golden Retriever, in which six inherited neurological disorders with a known causal variant have been documented, and the Belgian Shepherd, in which five such disorders have been documented. DNA tests can play a vital role in effectively managing and ultimately eradicating inherited diseases.

## 1. Introduction

Many hereditary neurological conditions have been documented in dogs. These conditions encompass congenital, neonatal, and late-onset disorders, along with both progressive and non-progressive forms. Consequently, it is possible for any dog with neurological clinical signs to have an underlying inherited disorder. In cases where the variant follows a recessive pattern, clinically affected animals are only born when there has been a history of inbreeding, leading to a dog inheriting two identical copies of the variant, one from each parent, which are both healthy. Consequently, specific inherited diseases are typically associated with particular breeds or closely related breeds due to their shared genetic heritage [1].

In order to identify the causal variant of a disease, the main two approaches are genome-wide investigations and candidate gene investigation. In a comprehensive genome-wide investigation, the entire genome is scrutinized to identify regions that are potentially harboring the variant(s) associated with a disease. This exploration can be conducted through pedigree-based linkage analysis, a genome-wide association study (GWAS), or whole genome sequencing [1]. A GWAS analyzes a great number of genetic variants across multiple genomes to identify statistical associations with specific traits or diseases. This approach has yielded numerous strong associations for various traits and disorders, and the count of linked variants is anticipated to expand with larger GWAS sample sizes [2].

Diseases in dogs are more prevalent in particular purebred breeds due to founder effects that have arisen from centuries of highly selective breeding practices. In dogs, where genetic diversity is lower and linkage disequilibrium is significantly higher compared with humans, the investigation of diseases within purebred populations serves as a robust approach for genomic mapping [3,4,5]. In 2005, the publication of the high-quality draft genome sequence of the domestic dog was accompanied by a comprehensive map of single nucleotide polymorphisms (SNPs) spanning various dog breeds [6].

Online Mendelian Inheritance in Animals (OMIA) serves as a compendium that documents inherited disorders and single-locus traits, as well as associated genes and variants across 484 different animal species. OMIA currently lists 418 Mendelian disorders specific to dogs, of which 355 have their likely causal genetic variant identified [7].

This review aims to summarize the current knowledge on the canine nervous system phenes and their genetic causal variant. Among them, categories are synthesized as follows: hereditary ataxias, lysosomal storage disorders, neuronal axonal dystrophy, hereditary neuropathies, congenital myasthenic syndromes, epilepsies, deafness, dyskinesias, encephalopathies and myelopathies, hypomyelinating disorders and leukodystrophies, neurometabolic disorders, and other inherited neurological conditions, which are presented below.

## 2. Canine Neurological Disorders with Known Causal Variants

### 2.1. Hereditary Ataxias

In humans, hereditary (cerebellar) ataxia encompasses a diverse range of neurodegenerative conditions that display variations in clinical signs, pathological features, and genetic origins. These disorders are characterized by the gradual deterioration of the cerebellum and, to varying extents, other structures outside the cerebellum [8]. The primary clinical characteristic of cerebellar ataxia becomes apparent through clinical signs such as unsteadiness and a loss of coordination. Ataxia can either be the exclusive indication of cerebellar impairment or, more commonly, be accompanied by a diverse array of other neurological signs [9].

In a prior review article conducted by Urkasemsin et al. [10] concerning hereditary ataxias in canines, five distinct categories were established based on neuropathological criteria: cerebellar cortical degenerations (CCDs), spinocerebellar degenerations, cerebellar ataxias with limited neurodegeneration, canine multiple system degeneration (CMSD), and episodic ataxia. These conditions are manifested across various dog breeds, each with differing onset ages, rates of advancement, combinations of clinical indicators, and specific locations of lesions within the central nervous system (CNS) [10,11].

Cerebellar ataxia typically results in symmetric hypermetria, a type of dysmetria, characterized by unimpeded flexor actions, especially during gait, leading to exaggerated limb lifting. In contrast, spinocerebellar ataxia is often described as having a more dance-like or bouncing quality [11,12].

Research on ataxias in Belgian Shepherd dogs identified a potential causative genetic variant in the *KCNJ10* gene linked to spongy degeneration with cerebellar ataxia, subtype 1. The study unveiled genetic diversity among dogs with similar clinical signs, implying the existence of multiple forms of cerebellar ataxia within the Belgian Shepherd breed. The onset of clinical signs was between 4.5 and 8.5 weeks of age, and the dogs exhibited an unsteady and irregular gait, characterized by a lack of coordination, particularly noticeable in the hind limbs. A notable 50% of the affected puppies displayed amplified and abnormal movements during locomotion. Additional inconsistent clinical manifestations included frequent stumbling, a staggering gait, unintentional shaking, and a hopping pattern reminiscent of a bunny, along with compromised equilibrium and instances of falling. The progression and severity of the clinical signs led to the puppies’ euthanasia by the 4th month of their life [13]. In Jack Russell terriers, a missense alteration in the gene (*KCNJ10*) responsible for the inwardly rectifying potassium channel Kir4.1 (c.627C>G) demonstrated a strong and statistically significant association with the condition [14].

An additional variant related to cerebellar ataxia was identified in Belgian Shepherd dogs. The likely causal variant involved a ~4.8 kilobase (kb) deletion in the critical interval, comprising exon 35 of the *RALGAPA1* gene [15]. Also, the same type of deletion is associated with atrophy of the CNS, particularly in the cerebellum. This deletion encompasses the entire sequence responsible for encoding the *SELENOP* protein and is anticipated to lead to the total absence of the selenoprotein P it encodes, which is essential for transporting selenium into the central nervous system [16].

Using a combined approach that combines linkage and homozygosity mapping, researchers pinpointed a critical region spanning approximately 10.6 megabases (Mb) on chromosome 5. This discovery emerged from the study of a Belgian Shepherd family where four puppies exhibited cerebellar dysfunction. Upon inspecting the 10.6 Mb interval in the whole-genome sequencing data from one affected puppy, a 227-base pair short interspersed nuclear element (SINE) insertion was identified within the *ATP1B2* gene. This SINE insertion resulted in abnormal RNA splicing. Immunohistochemistry findings indicated a decrease in ATP1B2 protein expression within the central nervous system of the affected puppies. The variant ATP1B2:c.130_131ins227 was, therefore, considered the most probable causative candidate for a second subtype of spongy degeneration with cerebellar ataxia in Belgian Shepherd dogs, now referred to as spongy degeneration with cerebellar ataxia subtype 2 (SDCA2) [17].

In Old English Sheepdogs and Gordon Setters with hereditary ataxia, a phenotype with onset between 6 months and 4 years of age was documented. Dogs exhibited prominent hypermetria, accompanied by a swaying of the trunk and intention tremors, with the symptoms progressively leading to significant gait disturbances. Cerebellar atrophy was detected through MRI. Targeted sequence capture and next-generation sequencing of the region identified an A to C single nucleotide polymorphism (SNP) located at position 113 in exon 1 of *RAB24*, a gene associated with autophagy [18].

An early onset progressive cerebellar ataxia was identified in the Norwegian Buhund, with signs including a wide-based stance and hypermetria in all limbs, alongside persistent head tremors and truncal ataxia. Whole-genome sequencing identified a T to C single nucleotide polymorphism (SNP) within the *KCNIP4* gene (g.88890674T>C), predicted to cause a tryptophan to arginine substitution in a highly conserved region of the potassium voltage-gated channel interacting protein KCNIP4 [19,20].

A similar early-onset phenotype, also known as Bandera’s neonatal ataxia, was documented in the Coton de Tulear dog breed. The puppies presented at two weeks of age with intention tremors, head titubation, and pronounced ataxia in gait, stance, and ocular movements. The disorder was mapped via a GWAS, and a retrotransposon inserted into exon 8 of gene *GRM1* (which encodes metabotropic glutamate receptor 1) was identified as a causal variant [21,22].

The analysis of early-onset progressive cerebellar ataxia in the Finnish Hound discovered a potential functional candidate gene, sel-1 suppressor of lin-12-like (*SEL1L*), and led to the discovery of a homozygous missense variant within a remarkably conserved protein domain. The affected puppies presented with swiftly progressing generalized cerebellar ataxia, tremors, and an inability to thrive. This variant is denoted as c.1972T>C, resulting in the amino acid change p.Ser658Pro [23].

Inherited ataxia in the Norwegian Elkhound, showing vestibulocerebellar signs with onset at 4 weeks of age and histologically distinguishable degenerations in the brain stem and cerebellum was associated with a 1 base pair (bp) deletion in the *HACE1* gene, which resulted in a frameshift at codon 333 and a premature stop codon at codon 366 [24].

Spinocerebellar ataxia, observed in the Parson Russell Terrier canine breed, is characterized by the onset of clinical signs, including gradual development of uncoordinated movement and a decline in balance, between 7 and 12 months of age. A single-nucleotide polymorphism (SNP) within the *CAPN1* gene is strongly associated with this pathology. The gene is responsible for encoding the calcium-dependent cysteine protease calpain1 (mu-calpain), and the variant leads to a substitution of cysteine with tyrosine at residue 115 of the CAPN1 protein [25].

In Italian Spinone dogs, there was a repetitive sequence expansion within intron 35 of the *ITPR1* gene, resulting in reduced protein expression in Purkinje cells. This occurrence was highly correlated with the development of spinocerebellar ataxia [26].

In Alpine Dachsbracke dogs, with clinical signs including clinical manifestations such as a wide-based stance, spinocerebellar ataxia distinguished by hypermetria in the thoracic limbs, hyperflexion in the pelvic limbs, compromised balance, pendular nystagmus, and truncal swaying, a specific genetic variant affecting a protein within the essential region of the *SCN8A* gene (c.4898G>T; p.Gly1633Val) has been linked to spinocerebellar ataxia. *SCN8A* encodes a voltage-gated sodium channel [27].

In Belgian Shepherd dogs, a truncating variant causing loss of function in the *SLC12A6* gene results in a phenotype associated with slowly progressing spinocerebellar ataxia, paraparesis, and muscle contractions resembling myokymia [28].

In Beagle dogs, neonatal cerebellar cortical degeneration associated with signs of progressive cerebellar ataxia is linked to an 8 bp deletion in the *SPTBN2* gene, which encodes β-III spectrin [29].

Cerebellar abiotrophy is marked by the degeneration of Purkinje and granule cells within the cerebellar cortex, and is observed in the Australian Working Kelpie dog breed. The clinical signs associated with cerebellar abiotrophy encompass ataxia, head tremors, motor coordination difficulties, a wide-based stance, and a distinctive high-stepping gait. The disease is associated with a missense variant in exon 5 of the vacuole membrane protein 1 (*VMP1*) gene [30].

Cerebellar cortical degeneration is a neurodegenerative disease that affects various dog breeds. The common presentation involves progressive cerebellar ataxia, with variations in the age of onset and progression rate among different breeds. In the Hungarian Vizsla, the clinical signs encompass pronounced hypermetric and dysmetric ataxia, swaying of the trunk, intentional tremors, the absence of menace responses, and positional horizontal nystagmus. The associated causal variant indicates an exon 26 splice donor variant in the sorting nexin 14 (*SNX14*) gene [31].

In Nova Scotia Duck Tolling Retrievers, a phenotype termed cerebellar degeneration—myositis complex with clinical signs of generalized ataxia and hypermetria, which was more pronounced in the pelvic limbs—was identified. The candidate causal variant was a missense (p.Pro446Leu) in the *SLC25A12* gene [32].

A Dandy–Walker-like malformation arises from abnormal brain development and is primarily characterized by cerebellar hypoplasia. Specifically, it involves the consistent absence of the rear parts of the cerebellar vermis and, to a lesser extent, the posterior portions of the cerebellar hemispheres, often accompanied by substantial fluid accumulation behind the cerebellum. Dogs affected by this condition exhibit non-progressive ataxia, with the severity of ataxia varying from mild signs, such as trunk swaying, subtle uncoordinated gait, balance issues, and hind limb ataxia, to severe cerebellar ataxia in puppies and episodes of rolling or falling. This condition in Eurasier dogs is associated with a single bp deletion (c.1713delC) in the *VLDLR* gene [33].

In Australian Shepherd dogs affected by hereditary ataxia, clinical signs consisted of moderate ataxia, particularly noticeable in the pelvic limbs. There was also a slight tendency toward overstepping (hypermetria) and minimal-to-no deficits in proprioception in the pelvic limbs. These signs indicated symmetric involvement of the cerebellum. The likely causal variant was a duplication (c.1169_1170dupTT) located in located in the *PNPLA8* gene [34].

An inherited neurodegenerative disorder was described in the Lagotto Romagnolo, which experienced a gradual onset of cerebellar ataxia, occasionally accompanied by intermittent nystagmus and alterations in behavior. The clinical entity was named neurodegenerative vacuolar storage disease, based on the histological examination, which showed extensive enlargement and clear formation of vacuoles within the neuronal cytoplasm, affecting both the central and peripheral nervous systems. The causal variant was identified as a missense change (c.1288G>A) in the autophagy-related *ATG4D* gene [35].

Canine multiple system degeneration (CMSD) is a progressive inherited neurodegenerative condition, typically marked by the degeneration and depletion of neurons in the cerebellum, olivary nuclei, substantia nigra, and caudate nuclei. Dogs that are affected by this condition appear normal until they reach the age of 3–6 months, at which point they begin to exhibit cerebellar ataxia. The ataxia worsens over time, leading to akinesia and severe postural instability, ultimately requiring euthanasia between 1 and 2 years of age [36,37]. This condition has been linked to two separate breed-specific autosomal recessive variants in the *SERAC1* gene. In the Kerry Blue Terrier, it involves a nonsense variant (c.1536G>A) and, in the Chinese Crested, a 4-base pair deletion (c.182+1_182+4del) [11].

### 2.2. Lysosomal Storage Disorders

Lysosomal storage disorders are hereditary ailments triggered by genetic defects within lysosomal structures. These defects lead to insufficiencies in one of three components: lysosomal enzymes, transmembrane proteins, or activator proteins. Consequently, there is an abnormal buildup of biomolecules within the lysosomes of certain cell types [38].

The neuronal ceroid lipofuscinoses (NCLs), also known as Batten disease, represent a set of inheritable lysosomal storage disorders marked by ongoing neurodegeneration, alongside the buildup of self-fluorescent lysosomal storage granules in the central nervous system and various other tissues. Individuals affected by these conditions experience vision impairment, gradual deterioration in both motor and cognitive functions, and episodes of seizures. Typically, the disease progresses to a state of continuous vegetative existence and early mortality [39]. Presently, there is no successful treatment available for any of the NCLs. 

In Dachshunds dogs affected by NCL type 1, the onset of clinical signs was at 7 months of age and the causal variant was found to be a single nucleotide insertion within exon 8 of the *PPT1* gene. This gene is responsible for encoding the enzyme palmitoyl protein thioesterase 1 [40]. Also, a case study reported a Cane Corso diagnosed with the same pathology but with a splice donor variant (c.124+1G>A) of the same gene [41].

NCL type 2 was also identified in Dachshunds with the same onset age as NCL type 1, and the variant responsible for the condition is a single nucleotide deletion (c.325delC) occurring in exon 4 of the *TPP1* gene. This deletion results in a frameshift of the amino acid codons and the premature emergence of a stop codon [42].

For NCL type 5, the onset age is between 1 and 2 years of age and the responsible variants have been identified in Border Collies, Australian Cattle dogs, and Golden Retrievers. For Border Collies and Australian Cattle dogs, a nonsense variant (c.619C>T) within exon 4 of the *CLN5* gene was identified as the causal variant [43,44]. In Golden Retrievers, a deletion (c.934_935delAG) within the same gene was predicted to cause a frameshift, resulting in a premature termination codon and the production of a protein variant lacking 39 C-terminal amino acids [45].

In Australian Shepherd dogs, NCL type 6 was linked to a missense variant involving a T to C variant (c.829T>C) located in exon 7 of the *CLN6* gene. This transition from T to C leads to an amino acid alteration, changing tryptophan to arginine in the predicted protein sequence [46].

In Chihuahuas and Chinese Crested dogs affected by NCL type 7, the underlying variant is a single-base pair deletion (c.843delT) found within the *MFSD8* gene. This deletion is expected to induce a frameshift and an early stop codon, ultimately leading to the production of a truncated protein lacking the last 239 C-terminal amino acids [47,48,49,50].

For NCL type 8, the onset of clinical signs is at 14–18 months and the causal variant was firstly identified in the English Setter and involves a T to C transition in the *CLN8* gene that predicts a p.L164P missense variant [51]. In the Alpine Dachsbracke, the causal variant involves the deletion of the entire CLN8 gene [52], while, in Salukis, a single bp insertion (c.349dupT) in exon 2 of the same gene is involved [53]. Additionally, in German Shorthaired Pointers and Australian Shepherds, a CLN8:c.585G>A transition in the same gene (*CLN8*) that predicts a CLN8:p.Trp195* nonsense variant was determined as the causal variant [54,55].

In the case of American Bulldogs suffering from NCL type 10, those affected were found to carry a homozygous A allele due to a G to A transition within the cathepsin D gene (*CTSD*). The onset of clinical signs was before the age of 2. This genetic alteration results in the conversion of methionine-199 to isoleucine [56].

In Tibetan Mastiffs with NCL type 12, the onset of clinical signs was at 4 to 6 years of age. The responsible variant was found in the canine *ATP13A2* gene. NCL-affected dogs were identified as homozygous for a single-base deletion within *ATP13A2*, which is expected to result in a frameshift and the emergence of a premature termination codon [57]. A c.1118C>T variant in the same gene that predicts a nonconservative p.(Thr373Ile) amino acid substitution was identified as causal for the same disease in Australian Cattle dogs [58]. 

In Dalmatians, a progressive neurological condition was documented. The onset was at around 1.5 years of age, and included heightened anxiety, increased sensitivity to stimuli, cognitive decline, disrupted sleep patterns, diminished coordination, and urinary and fecal incontinence, as well as visual impairments. The age of onset and the severity varied among individuals. Intracellular inclusions displaying autofluorescence were identified in the cerebral cortex, cerebellum, optic nerve, and cardiac muscle, concurrently correlating with immunolabeling of the lysosomal marker protein LAMP2 and binding of antibodies to mitochondrial ATPase subunit c. The likely causal variant was a single-base deletion and frameshift near the 3′-end of the *CNP* gene [59].

The mucopolysaccharidoses (MPS) constitute a varied collection of lysosomal storage diseases distinguished by the inadequate function of specific lysosomal enzymes vital for the complete degradation of glycosaminoglycans [60]. For MPS type I, the clinical manifestations include stunted growth, the development of degenerative joint ailments, pronounced bone abnormalities (dysostosis multiplex), cardiac complications, and impaired vision, attributable to the presence of corneal clouding. The specific enzymatic modification is a marked deficiency of the alpha-L-iduronidase activity [61]. In the Plott Hound, a G to A transition in the donor splice site of intron 1 of the *IDUA* gene was identified as the causal variant [62]. Variants within the same *IDUA* gene are also incriminated in the occurrence of MPS type I in Boston Terriers (an 8-nucleotide insertion) and in Golden Retriever (a 287 bp deletion resulting in full deletion of exon 10) [63,64].

MPS type IIIA is a hereditary disorder inherited in an autosomal recessive manner, stemming from a deficiency in heparan sulfate sulfamidase [65]. The disorder was identified in Dachshunds and New Zealand Huntaway dogs. In Dachshunds, the onset age was 3 years and the dogs displayed pelvic limb ataxia, which gradually intensified over the course of 1 to 2 years, leading to pronounced and widespread spinocerebellar ataxia [66]. The causal variant was identified as a 3-bp deletion in the *SGSH* gene [67]. In New Zealand Huntaway dogs, a more severe phenotype was documented, in which the disease onset occurred at approximately 1.5 years of age and swiftly progressed over a period of one month, resulting in profound ataxia [68]. The causal variant was identified as a single bp insertion in the same *SGSH* gene [65].

MPS type IIIB was identified in the Schipperke dog breed. The clinical signs were observed at approximately 3 years of age and were consistent with cerebellar disease, including hind limb ataxia, dysmetria, and a broad-based stance accompanied by trunk swaying. Mildly dystrophic corneas and small peripheral areas of retinal degeneration were also observed. MPS type III is characterized by a progressive nature that ultimately necessitates humane euthanasia before the age of six. Deficient enzymatic activity was noticed for the lysosomal glycosidase N-acetyl-alpha-D-glucosaminidase [69]. The causal variant was identified within exon 6, characterized by an insertion composed of a 40–70 bp poly-A and an 11 bp duplication of the exonic region preceding the poly-A within the *NAGLU* gene [70].

MPS type VI is induced by decreased or absent activity of the lysosomal enzyme N-acetylgalactosamine 4-sulfatase (or arylsulfatase B) [71]. In Miniature-Poodle-like dogs, the presentation for clinical examination was at 2.5 years, with signs of gross joint laxity and the development of bilateral corneal opacities. Radiological assessment identified malformed vertebral bodies, widespread epiphyseal dysplasia, and luxation/subluxation of the lumbosacral and appendicular joints, accompanied by underdevelopment of the hyoid apparatus and odontoid process. The degenerative nature of the disorder led to euthanasia by the age of 3, due to the decreasing quality of life. A 22 bp deletion in exon 1 of the *ARSB* gene was pinpointed as the causal variant [72]. In Great Danes with similar clinical signs but with their onset at 4 months, a nonsense variant at position 295 in exon 1 of the same gene (*ARSB*) was identified [73]. In Miniature Pinschers and Miniature Schnauzers with MPS type VI, two different types of variants were identified in the *ARSB* gene: a 56 bp deletion at the junction of exon 1 (for the Miniature Schnauzers) and a missense variant in exon 5 (for the Miniature Pinschers) [74].

MPS type VII is an hereditary disorder caused by a deficiency in the activity of the lysosomal acid hydrolase beta-glucuronidase [75]. A case report documented MPS type VII in a German Shepherd, with onset of the clinical signs at the age of 3 months. Signs included disproportionate dwarfism, excessive joint flexibility, muscular atrophy of the limbs, and bilateral cloudy corneas with diffuse granularities. A missense variant at nucleotide 599 within the *GUSB* gene was identified as the causal variant [76]. In Brazilian Terriers, the affected puppies exhibited profound skeletal deformities, noticeable during the initial month of life. Radiographic imaging and histology revealed delayed ossification and spondyloepiphyseal dysplasia. The progressive nature of the disease let to the euthanasia of the affected dogs. The causal variant was a missense variant (c.866C>T) of the same *GUSB* gene [77].

A late onset form of mucopolysaccharidosis associated with reduced arylsulfatase activity was identified in an American Staffordshire Terrier. The onset of the disorder was between the ages of 3 and 5 years, with signs of ataxia and no visual impairment specific to other NCLs. MRI and necropsy examination revealed cerebellar atrophy. The causal variant was identified as a G to A substitution in exon 2 of the *ARSG* gene [78,79].

Alpha-mannosidosis represents an inherited lysosomal storage disorder distinguished by immune deficiency, facial and skeletal irregularities, hearing impairment, and cognitive disability [80]. The disorder was identified in a Doberman Pinscher at two months of age, and included an unusually broad calvarium, protrusive frontal sinuses, proprioceptive ataxia, dulled cognitive function, and moderate divergent strabismus. As the disease progressed, the dog was euthanized at 14 months of age due to indications of dementia and evident hallucinatory behavior. A deficiency in the alpha-mannosidase enzyme activity was detected, together with specific autofluorescent vacuolar inclusions. The causal variant was a missense variant (Asp104Gly) in the *MAN2B1* gene [81].

Beta-mannosidosis denotes an inherited anomaly within glycoprotein catabolism [82]. In a German Shepherd, the onset of the disorder was noticed at 2 months of age, with signs such as slow growth, retained deciduous teeth, and deafness. At 8 months of age, euthanasia was decided due to the stiffness and weakness in the neck and hind limbs, proprioceptive deficits in all four limbs, and depressed mental status. The specific deficiency in beta-mannosidase activity was also detected. The identified causal genetic variant was a T to A transition occurring within exon 4 of the *MANBA* gene [83]. A different variant was identified in a mixed breed dog with beta-mannosidosis consisting of five bp tandem duplication in exon 16 of the same *MANBA* gene [84].

GM1 gangliosidosis is a degenerative neurosomatic lysosomal storage disorder attributed to variants in the *GLB1* gene, which encodes the enzyme β-galactosidase [85]. The onset age is around 5 months, and dogs affected by the condition typically exhibit dwarfism and advancing cerebellar impairment, together with limb weakness. Manifestations encompass weight loss, ataxia, a broad-based gait, diminished proprioception, intention tremors of the head, hypermetria, dysmetria, internal strabismus, and positional nystagmus. A loss of β-galactosidase activity is also noted [86,87]. In Portuguese Water Dogs, the identified variant was a G to A transition in the exon 2 of the *GLB1* gene, while, in the Shiba Inu and Miniature Shiba Inu, the identified variant was a deletion of a single bp (cytosine) in exon 15 of the same *GLB1* gene [88,89,90]. In the Alaskan Husky, the causal variant associated with GM1 gangliosidosis was a 19 bp insertion (duplication) in exon 15 of the *GLB1* gene [91].

The GM2 gangliosidoses refer to a collection of lysosomal storage disorders distinguished by the accumulation of GM2 ganglioside and related glycolipids, primarily within neurons. In Japanese Chin dogs, the onset age of the clinical signs in individuals with GM2 gangliosidosis type I was approximately 18 months of age, and included gradually worsening cerebellar ataxia, alterations in mental state, and visual impairment. An MRI of the brain showed diffuse atrophy, and an increase in beta-hexosaminidase was noticed. The degenerative nature of the disorder culminates in either death or necessitates euthanasia within a few months. The causal variant for this disorder in Japanese Chin dogs was pinpointed to a G to A transition in exon 8 of the *HEXA* gene [92]. Similar clinical signs were noted in GM2 gangliosidosis type II (or variant 0), but with an earlier onset by the age of 1, and occurrence of death by the age of 2. Additionally, a significant decrease in beta-hexosaminidase activity was identified. A single bp deletion of guanine in exon 3 of the *HEXB* gene was associated with the disorder in the Toy Poodle, while, in the Shiba Inu, the causal variant was a 3 bp deletion within the same gene [93,94].

Glycogen storage disease type II (also known as Pompe disease in humans) is an autosomal recessive lysosomal storage disorder that arises from the deficiency of acid alpha-glucosidase. The disorder was identified in Finnish and Swedish Lapphunds, with onset of clinical signs at around 6 months of age, and included severe vomiting, together with marked developing muscle weakness. As the disease progressed, constant panting, difficulty in breathing, altered vocalization, and difficulties in swallowing were evident, as a consequence of the myocardial hypertrophy and esophageal dilatation. The characteristic severe deficiency in acid alpha-glucosidase was noted. Affected dogs usually survive to the age of 2 years [95,96]. A missense (c.2237G>A) within *GAA* gene, which results in a premature stop codon, was identified as the causal variant [96].

Alpha fucosidosis, a lysosomal storage disorder, emerges due to the deficiency of the lysosomal enzyme alpha-L-fucosidase, resulting in the accumulation of undegraded fucose-rich substances within various organs [97]. The disorder is documented in the English Springer Spaniel, occurring at the age of 6 months with behavioral changes. As the disease advances, signs include gradually worsening ataxia, impaired proprioception, alterations in temperament, difficulties in swallowing, altered vocalization, loss of acquired behaviors, muscle atrophy, and apparent visual impairment. Typically, affected dogs succumb to the illness or require euthanasia by the age of 3 to 4 years. The causal variant in affected dogs was pinpointed to a 14-base pair deletion at the 3′ end of exon 1 in the *FUCA1* gene [98]. 

Krabbe disease is an autosomal recessive condition that arises due to an insufficiency in galactocerebrosidase activity. In dogs, clinical sign occurs between 4 and 6 weeks of age. Dogs affected by the condition typically exhibit initial signs of tremors and weakness in the pelvic limbs, which subsequently advance to pelvic limb ataxia and dysmetria in the thoracic limbs. This is succeeded by the onset of tetraparesis and, ultimately, paralysis in the hind limbs between the ages of 3 to 5 months, requiring euthanasia [99]. In the Cairn Terrier and West Highland White Terrier, a base substitution (A to C transversion) at position 473 of the complementary DNA for the *GALC* gene was identified as the causal variant [100]. In the Irish Setter, a 78 bp insertion within the same gene was pinpointed as the causal variant [101]. Also, in a cohort of mixed breed puppies, a missense variant in the *GALC* gene was the likely cause of Krabbe disease [102].

### 2.3. Neuronal Axonal Dystrophy

Neuroaxonal dystrophy (NAD) is a neurodegenerative condition with a distinct histological presentation that can affect either the central or peripheral nervous system, and that lacks specificity in its clinical presentation. NAD is identified by the presence of localized axonal swellings, known as spheroids, and axonal atrophy. Puppies with this condition typically display fetal akinesia, scoliosis, arthrogryposis, cerebellar hypoplasia, pulmonary hypoplasia, thinning of the patellar tendon, spinal cord hypoplasia, and, ultimately, experience respiratory failure [103].

For NAD, the causal variant has been identified in a few dog breeds. In Papillon dogs, the c.1579G>A missense variant in the *PLA2G6* gene has been associated with the presence of this pathology [104]. A missense variant (g.14777774T > C; p.H835R) is also responsible for the NAD phenotype in young adult Rottweiler dogs, yet the variant is located in the vacuolar protein sorting 11 (*VPS11*) gene [105]. Additionally, in Spanish Water Dogs, juvenile-onset NAD was associated with a missense variant (c.4009C>T or p.R1337W) in the canine tectonin beta-propeller repeat-containing protein 2 (*TECPR2*) gene [106]. Other than the above-mentioned missense variants, a 3-nucleotide deletion in exon 13 of mitofusin 2 (*MFN2*) was associated with fetal-onset NAD in a breeding colony maintained at Michigan State University [107].

### 2.4. Hereditary Neuropathies

The clinical manifestations of sensory or sensorimotor polyneuropathies vary in accordance with the dysfunctions of the affected nerves. Canine inherited polyneuropathies are homologous to Charcot–Marie–Tooth (CMT) disease in humans, sharing similar clinical signs and responsible gene variants. It is important to highlight that inherited sensory and autonomic neuropathies are less prevalent than inherited motor and sensory neuropathies [108,109].

In mixed breed dogs with an absence of superficial and deep pain perception in the entire body, a missense variant (p.Arg921Cys) in the *SCN9A* gene was associated with this pathology [110].

Sensory ataxic neuropathy (SAN) is a neurological condition observed in Golden Retrievers. SAN typically begins subtly during puppyhood and advances gradually. Both males and females are equally affected. Dogs with SAN exhibit signs of ataxia, deficits in postural reactions, and diminished or completely absent spinal reflexes. A ΔT5304 deletion in the mitochondrially encoded tRNA tyrosine (*MT-TY* or *tRNA^Tyr^*) gene was identified as a causal variant [111].

In mixed breed dogs with chronic progressive proprioceptive ataxia manifesting as generalized muscle atrophy, mainly of the pelvic limbs, spontaneous knuckling, and self-mutilating wounds on the distal part of the pelvic limbs, a missense variant (c.656C > T, p.P219L) in the reticulophagy regulator 1 (*RETREG1* or *FAM134B*) gene was pinpointed as the plausible causal variant [112]. In Border Collies with similar symptomatology, an inversion within the same gene (*FAM134B*) was identified as a causal variant [113].

For acral mutilation syndrome, clinical signs manifest early in young puppies and typically include insensitivity to pain in the extremities, sometimes accompanied by sudden, intense biting, licking, and severe self-inflicted injuries to the feet. Proprioception, motor skills, and reflexes of the spinal cord remain unaffected. For this syndrome, a variant that alters the binding of the regulatory complex located 90 kb upstream of the *GDNF* gene was identified as a causal variant in German short-haired Pointers, English Pointers, English Springer Spaniels, and French Spaniels [114].

A hereditary polyneuropathy has been identified in Leonberger and Saint Bernard dogs. This condition is a severe, juvenile-onset, chronic, and progressive mixed polyneuropathy. It is marked by clinical signs such as exercise intolerance, irregular gait, and muscle wasting in the hind limbs, as well as inspiratory stridor and breathing difficulties. A variant in the *ARHGEF10* gene was pinpointed as causal and its sequencing revealed a 10-base pair deletion in dogs with this condition. This deletion encompasses four nucleotides from the 3′-end of exon 17 and six nucleotides from the 5′-end of intron 17 [115]. In Leonberger dogs, an additional association between polyneuropathies and a 2 bp deletion in the *GJA9* gene was confirmed [116].

In Miniature Schnauzers affected by demyelinating motor and sensory neuropathy, a 40-base pair deletion was discovered at the 3′ end of exon 19 within the *SBF2* gene as the causal variant. This deletion results in a premature stop codon, leading to a truncation of the protein by 1070 amino acids [117].

Polyneuropathy in the juvenile Greyhound was associated with a 10 bp deletion in exon 15 of the canine *NDRG1* gene [118]. Additionally, early-onset progressive polyneuropathy in Alaskan Malamutes was linked to a non-synonymous variant (G>T) occurring in exon 4 of the of the same (*NDRG1*) gene. This variant results in an amino acid substitution, changing glycine to valine at position 98 [119].

In Black Russian Terriers with polyneuropathy with ocular abnormalities and neuronal vacuolation, a single nucleotide deletion (c.743delC) within the *RAB3GAP1* gene was pinpointed as the causal variant [120]. The same variant was identified in Rottweilers suffering from neuronal vacuolation and spinocerebellar degeneration [121]. The same gene (*RAB3GAP1*) is involved in the occurrence of polyneuropathy with ocular abnormalities and neuronal vacuolation in Alaskan Huskies, but the causal variant is a 218 bp SINE insertion into exon 7 [122].

Another entity known as laryngeal paralysis and polyneuropathy features dyspnea and stridor, dysphagia, dysphonia, high-stepping and uncoordinated gait, exercise intolerance, stumbling and tripping, and limb muscle atrophy. A missense variant (p.Gly937Glu) in the *CNTNAP1* gene was associated with laryngeal paralysis and polyneuropathy in the Leonberger, Saint Bernard, and Labrador Retriever [123].

### 2.5. Congenital Myasthenic Syndromes 

Myasthenia gravis is a condition impacting neuromuscular communication in dogs and cats. It has traditionally been classified into an acquired or autoimmune form, marked by autoantibodies targeting the neuromuscular junction and typically manifesting after 6 months of age, and a congenital form, which lacks neuromuscular junction autoimmunity and tends to manifest within the first few weeks to months of life. Congenital syndromes affecting the neuromuscular junction, resulting in muscle weakness and fatigue, are collectively referred to as congenital myasthenic syndromes. The term myasthenia gravis exclusively pertains to autoimmunity against the neuromuscular junction [124].

In Jack Russel Terriers with congenital myasthenic syndromes manifested as generalized muscle weakness at 7 weeks of age, the causal variant was identified as a single-base insertion occurring in exon 7 of the *CHRNE* gene. This insertion results in a frameshift variant and the appearance of a premature stop codon [125]. A similar single-base variant within the same gene (*CHRNE*) was identified as the plausible candidate in the Heideterrier, with lack of reflexes and coordination of the front limbs [126]. In Old Danish Pointer dogs with congenital myasthenic syndrome, a G to A missense variant in exon 6 of the gene encoding choline acetyltransferase (*CHAT*) was identified as possibly causal [127].

### 2.6. Epilepsies

According to the International League Against Epilepsy, an epileptic seizure is expounded as the temporary manifestation of signs arising from irregular and heightened neuronal activity in the brain, while epilepsy is a brain disorder distinguished by a persistent inclination to experiencing an epileptic seizure. This description of epilepsy mandates the presence of a minimum of one epileptic seizure for classification. This particular definition of epilepsy mandates the presence of at least one epileptic seizure, although one could argue that, for a comprehensive diagnosis of epilepsy, it is necessary for the individual to undergo two distinct occurrences of seizures, indicating a repeated pattern [128,129].

A type of generalized myoclonic epilepsy with photosensitivity in young dogs was identified in the Rhodesian Ridgeback breed. The mean age of onset age of clinical signs was 6 months, based on the study of 95 individuals. The myoclonic twitches predominantly manifested when the dogs were lying down, in a state of relaxation, drowsiness, or during initial phases of sleep. On occasion, these twitches also emerged when the dogs were in a seated, standing, or ambulatory posture. The progression from myoclonic seizures to generalized tonic–clonic seizures was observed in 38% of dogs within 6 months after the onset. The diagnostic examinations did not reveal any uniform structural irregularities. Visual stimulus-induced seizures were recorded in 35% of the Rhodesian Ridgebacks with generalized myoclonic epilepsy. The causal variation involves a 4-bp deletion in the exon 2 of the *DIRAS1* gene [130].

Another phenotype of epilepsy known as benign familial juvenile epilepsy (or remitting focal epilepsy) was identified in the Lagotto Romagnolo. The onset of seizures occurs during early life, typically between five and nine weeks of age, with spontaneous remission by thirteen weeks of age. Afflicted puppies show generalized tremors, lack of coordination, and stiffness during seizures, while severe cases may exhibit additional neurological signs such as unsteady gait and excessive muscle activity between epileptic episodes. BFJE puppies exhibit epileptiform activity on electroencephalogram, both during and between epileptic episodes. The associated causal variant concerns the truncation (c.1552A>T) of the *LGI2* gene, which prevents secretion and action on neuronal ADAM receptors [131,132].

Lafora disease (myoclonus epilepsy of Lafora) is an autosomal recessive genetic disorder identified by the buildup of polyglucosan bodies, also known as Lafora bodies, within the perikaryon and dendrites of neurons in the central nervous system. This condition results in the gradual onset of neurological impairments, predominantly recognized by the presence of myoclonic epilepsy [133]. Lafora disease is characterized by progressive myoclonic epilepsy with a late onset of the clinical signs (8.3 years, median for Beagles) and initial clinical manifestation consisting of myoclonic episodes, accompanied by episodes of generalized tonic–clonic seizures [134]. The causal variation was first identified in the Miniature Wirehaired Dachshund and involved a range of 19 to 26 copies of a 12-nucleotide sequence (dodecamer) within the canine *NHLRC1* gene (formerly *EPM2B*) [135]. The same variation was also identified in other canine breeds diagnosed with Lafora disease such as the Newfoundland [136], Chihuahua [137], and Pembroke Welsh Corgi [138].

A particular form of epilepsy was documented in the Parson Russell Terrier (epilepsy with mitochondrial dysfunction and neurodegeneration). The disease commenced with epileptic seizures typically emerging between 6 and 12 weeks of age and rapidly advanced to a state of status epilepticus, culminating in death or necessitating euthanasia. Histological analysis unveiled an occurrence of acute neuronal degeneration and necrosis, diffusely impacting the grey matter across the entire brain, accompanied by accumulation of amyloid-β and substantial intraneuronal crowding of mitochondria. The pinpointed causal variant was a 6-bp deletion in the *PITRM1* gene [139].

### 2.7. Deafness

The ear serves as a highly intricate sensory apparatus accountable for hearing, as well as the vestibular regulation of posture and ocular movements. Disorders affecting the inner ear are prevalent among dogs and cats, frequently linked with neurological impairment such as deafness, peripheral vestibular syndrome, Horner’s syndrome, and facial paralysis [140].

The occurrence of congenital deafness is widespread across dog breeds. In Australian Stumpy Tail Cattle Dogs, a missense variant of the *KLF7* gene was associated with deafness in this breed [141]. In the Beauceron breed, bilateral hearing loss was associated with a missense variant in the *CDH23* gene [142]. A particular type of early onset adult deafness was identified in Rhodesian Ridgebacks, with occurrence within the first 1–2 years of life. The associated variant was a 12 bp deletion in the *EPS8L2* gene [143]. In Rottweilers, sensorineural bilateral deafness with onset at a few weeks of age was associated with a missense variant in the *LOXHD1* gene [144].

A particular phenotype of deafness also includes vestibular deficits. This phenotype was identified in the Doberman Pinscher breed, with onset of the vestibular deficits between birth and 10 weeks of age [145]. In the case of affected Doberman Pinscher puppies, clinical observations confirmed the presence of both bilateral vestibular dysfunction and bilateral deafness. As the puppies commenced their early locomotor activities and nursing behaviors, the vestibular dysfunction became evident through conspicuous side-to-side movements of the head and neck, frequent instances of body collapse, a characteristic “bobble-head” appearance, and intermittent head tilting. Notably, the puppies exhibited an absence of startle responses, and brainstem auditory evoked response tests indicated complete sensorineural deafness. Subsequent histological examinations of inner ear tissue confirmed cochlear anomalies, revealing a complete degeneration of the organ of Corti without involvement of the stria vascularis, indicative of consistent neuroepithelial degeneration. The likely causal variant was identified as a missense (c.3719G>A) variant in the *MYO7A* gene [146]. Also, in the Doberman Pinscher, a slightly different phenotype was documented, with dysfunction of the peripheral vestibular system and unilateral deafness caused by a single-base insertion in the exon 39 of the *PTPRQ* gene [147].

### 2.8. Dyskinesias

Movement disorders encompass a diverse range of conditions found in both humans and animals, marked by involuntary movements that occur without alterations in consciousness. During an episode, the affected dogs do not display signs of autonomic dysfunction, show abnormalities in electroencephalographic patterns, or undergo changes in consciousness. In the field of veterinary medicine, the term ‘paroxysmal dyskinesia’ is employed as a broad phrase to refer to the recurring, sudden, and involuntary contraction of a set of skeletal muscles [10]. In Soft-Coated Wheaten Terriers with paroxysmal dyskinesia, having incidents of hyperkinesia and dystonia that persisted from several minutes to several hours, the possible causal variant was identified as a transition (c.398C>T) in the *PIGN* gene [148]. In Markiesje dogs, a juvenile form of paroxysmal dyskinesia was described. The affected puppies displayed severe clinical signs, including weakness in all four limbs, dystonia, muscle cramps, and stumbling or falling over when attempting to walk. The plausible causal variant was identified as an indel variant in the *SOD1* gene, in which a G-nucleotide of the fourth codon of the gene is replaced by a CAC-trinucleotide [149].

Hyperekplexia (startle disease) is a neurological disorder with onset at birth, in which puppies exhibit involuntary ‘startle responses’ characterized by widespread or sporadic stiffening of their limbs, triggered by sudden handling or loud noises. In the Irish Wolfhound, the affected puppies also experienced cyanosis and pneumonia while feeding, resulting from prolonged episodes of stiffness that lead to apnea. The causal variant in the Irish Wolfhound concerns a 4.2 kb microdeletion encompassing exons 2 and 3 in the *SLC6A5* gene [150]. In the Spanish Greyhound, a two-base pair deletion in exon 9 of the same gene (*SLC6A5*) was identified as the causal variant [151]. In the Miniature Australian Shepherd with clinical signs resembling hyperekplexia, the causal variant pinpointed a 36-bp deletion spanning the exon–intron boundary in the glycine receptor alpha 1 (*GLRA1*) gene [152].

Weimaraner dogs have been documented to exhibit a syndrome characterized by paroxysmal dystonia–ataxia. During these episodes, affected dogs displayed an abnormal gait marked by dystonia, ataxia, and hypermetria, sometimes leading to occasional collapses. Additional consistent features included kyphosis and holding the head low. The onset of signs occurred between 3 and 7 months of age. Emotional arousal or physical activity was noted as causal for these episodes, and could occur multiple times daily, lasting between 5 and 15 min. A frameshift variant (c.831dupC) in the *TNR* gene was identified as the plausible causal variant [153].

Episodic falling syndrome is a condition of sudden, temporary muscle stiffness observed in Cavalier King Charles Spaniels. These episodes are typically triggered by exercise, stress, or excitement, and are characterized by increasing muscle stiffness in the front and hind limbs, leading to a distinct ‘deer-stalking’ posture and possible collapse. The syndrome is a paroxysmal hypertonicity disorder, and in Cavalier King Charles Spaniels the plausible causal variant pinpoints a 15.7 kb deletion in the *BCAN* gene [154]. 

### 2.9. Encephalopathies and Myelopathies

Neonatal encephalopathy with seizures is an inherited disease specific to Standard Poodles. Puppies affected by this condition are born small and do not undergo normal development. At around 3 weeks of age, they exhibit signs including weakness, ataxia, tremors throughout their bodies, a wide-based stance with increased muscle stiffness, and weakness in their core muscles leading to neck ventroflexion. At around 3 to 6 weeks of age, they experience generalized clonic–tonic seizures and eventually become recumbent on their sides with rigid extensions and opisthotonus posture. Most of the puppies do not survive beyond 7 weeks of age. The causal variant is a missense c.152T>G transversion in the *ATF2* gene [155].

Subacute necrotizing encephalopathy was described in the Alaskan Husky and Yorkshire Terrier. Clinical signs have an acute onset and include multifocal central nervous system deficits such as altered mentation, seizures, absent menace response, central blindness, dysphagia, hypermetria, proprioceptive positioning deficits, ataxia, tetraparesis, and facial hypoalgesia. For the Alaskan Husky, the likely causal variant was a 4 bp insertion (c.624insTTGC) and SNP (c.625C>A) in the exon 2 of the *SLC19A3* gene, while, for the Yorkshire Terrier, the indel affecting ~45 bp was located in exon 2 of the same gene [156,157].

Hereditary necrotizing myelopathy is characterized by progressive ataxia and paralysis. The disease was described in the Dutch Kooiker, which exhibited exaggerated spinal reflexes and postural deficits in the hind limbs. The causal variant was identified in the *IBA57* gene associated with the iron–sulfur cluster assembly, resulting in an amino acid substitution known as R147W [158].

Canine degenerative myelopathy is a deadly neurodegenerative disease that occurs in adulthood and shares numerous similarities with an upper-motor-neuron-onset variant of human amyotrophic lateral sclerosis. The initial degeneration of upper motor neurons results in spastic paraparesis, and, as the condition progresses, affected dogs develop general proprioceptive ataxia in their hind limbs. As the disease advances, lower motor neuron signs become apparent, including ascending tetraparesis, flaccid paralysis, and widespread muscle atrophy [159]. The first identified potential causative variant was a G to A transition (c.118G>A) located in exon 2 of the *SOD1* gene. This variant was documented in more than 120 dog breeds [160]. The responsible variant was exclusively detected in Bernese Mountain dogs and involves a c.52A>T transition within the SOD1 gene. This variant leads to the substitution of threonine with serine at position 18 in the amino acid sequence [161].

### 2.10. Leukodystrophies and Hypomyelinating Disorders 

In Golden Retrievers with congenital hypomyelinating polyneuropathy, variants in three genes were identified as likely causal. The first variant concerns the MTMR2 (myotubulin-related protein 2) gene and is a G to A transition, located within the splice site at the end of exon 12. The second variant involves the *MPZ* (myelin protein zero) gene and is a missense located in exon 3. The third variant is a C to T nonsense variant in exon 11 of the *SH3TC2* (SH3 domain and tetratricopeptide repeats 2) gene [162].

Hypomyelination of the central nervous system was first described in the Chow-Chow. The affected dogs exhibited a wide-based stance in their hind limbs and displayed a ‘rocking-horse’ motion of their entire bodies when trying to walk. Hypermetria was noticeably pronounced in all of their limbs, and they adopted a ‘rabbit-hopping’ gait when walking on their hind limbs [163]. In affected Weimaraners, the distinctive abnormality observed was the presence of dysmyelination in the peripheral region of the spinal cord, particularly prominent in the cervical and thoracic levels. The variant responsible for the condition in Weimaraners was identified as a deletion of a single A nucleotide within exon 9 of the gene that codes for folliculin-interacting protein 2 (*FNIP2*) [164].

For X-linked tremor, the affected puppies may have a smaller size compared with their littermates and commonly display challenges in standing, along with ataxia and widespread tremors that typically manifest at around 10–12 days of age. The affected dogs often do not survive past 3–4 months of age. Significant hypomyelination is evident throughout the central nervous system, with more pronounced effects observed in the cerebrum and optic nerves, compared with the spinal cord [165]. This medical condition was described in the Springer Spaniel, and the variant responsible for the condition is a point variant occurring at position 219 within exon 2 of the coding sequence of the *PLP1* gene, leading to a substitution of histidine with proline in the protein [166].

The leukodystrophies represent a cluster of hereditary disorders affecting the white matter, characterized by a diverse genetic basis, substantial phenotypic diversity, and a disease onset at all ages [167].

Canine inherited spongiform leukoencephalomyelopathy (or leukodystrophy) is characterized by widespread vacuolation in the white matter of the brain and spinal cord. In Australian Cattle Dogs and Shetland Sheepdogs, the individuals affected by this condition exhibited tremors starting at 2–9 weeks of age, followed by a progressive neurological deterioration that included ataxia, muscle weakness, paralysis, spasticity, and dysfunction of cranial nerves. The entity is associated with a G to A transition (c.14474G>A) in the mitochondrial gene for cytochrome b (*CYTB*) [168]. In Standard Schnauzers with leukodystrophy, a missense variant affecting exon 5 of the *TSEN54* gene was identified as the likely causal variant [169]. In Great Danes and Rottweilers with leukoencephalomyelopathy, a 1 bp insertion (c.345_346insC) in the NAPEPLD gene was pinpointed as the causal variant, while, in Leonbergers, a missense variant (c.538G>C) within the same gene (*NAPEPLD*) was identified as causal [170].

Alexander disease, also part of the leukodystrophies group, is a deadly neurodegenerative disorder attributed to dysfunction of astrocytes in humans. In juvenile Labrador Retrievers, the clinical features included tetraparesis with spastic contraction of the anterior limbs resembling ‘swimming puppy syndrome’, and the pathological exam revealed the detection in astrocytes of *GFAP* containing Rosenthal fibers. The causal variant was identified as a nucleotide substitution (c.719G>A) in the *GFAP* gene [171].

### 2.11. Neurometabolic Disorders and Other Inherited Neurological Conditions

L-2-hydroxyglutaric aciduria in dogs is an autosomal recessive metabolic disorder characterized by a range of progressive neurological signs. These clinical signs include seizures, dementia, head tremors, muscle stiffness, and cerebellar ataxia, which involves a wide-based stance, swaying of the trunk, loss of balance, and uncoordinated gait. The distinctive biochemical feature of L-2-hydroxyglutaric aciduria is the buildup of L-2-hydroxyglutaric acid in cerebrospinal fluid, plasma, and urine. In Yorkshire Terriers, the disease was associated with a variant (c.1A>G) disrupting the translation of the initiation codon of the *L2HGDH* gene [172], while, in Staffordshire Bull Terriers, the causal variant involves a dual amino acid substitution occurring in exon 10 of the same gene, which consists of two single-nucleotide substitutions separated by a single unchanging T nucleotide [173].

Succinic semialdehyde dehydrogenase deficiency is a genetic disorder causing abnormal metabolism of the neurotransmitter γ-amino butyric acid (GABA) [174]. The clinical entity was described in Saluki puppies with neurological issues such as seizures and changes in behavior. Magnetic resonance imaging revealed a widespread and significant decrease in the thickness of the cerebral cortex, along with symmetrical areas of increased signal intensity in specific brain regions. Upon necropsy, cerebral cortical atrophy with vacuolation (status spongiosus) was observed. A missense variant (c.866G>A) was pinpointed as the causal variant, located within the *ALDH5A1* gene [175].

Medium-chain Acyl-CoA dehydrogenase deficiency is the most prevalent inherited metabolic disorder associated with the β-oxidation process [176]. The condition was observed in Cavalier King Charles Spaniels, which exhibited complex focal seizures characterized by extended periods of lethargy, reduced responsiveness, and proprioceptive ataxia. Brain MRI scans showed breed-specific changes, including occipital malformation with mild cerebellar herniation, syringohydromyelia, and medullary twisting, which are indicative of canine Chiari-like malformation and syringomyelia. Organic acid analysis of urine revealed significant excretion of hexanoylglycine and a suberic acid peak. The causal variant was identified as a private protein-changing variant (delins) in the *ACADM* gene [177].

Cerebellar hypoplasia is a characteristic seen in various neurological conditions where the cerebellum does not fully develop. In White Swiss Shepherds, this condition was observed in puppies that did not gain weight and experienced gradually worsening ataxia, starting at approximately 2 weeks of age. Postmortem examination showed severe cerebellar hypoplasia with lissencephaly. A frameshift deletion (p.Val947*) of the reelin (*RELN*) gene was pinpointed as the plausible causal variant [178]. 

Dilute coat color with neurological defects is the canine equivalent of Griscelli syndrome in humans. This condition was observed in Miniature Dachshunds that exhibited a visible coat color dilution and experienced difficulties holding their heads independently or maintaining a stable prone position for an extended duration. The candidate causative variant was an frameshift insertion (c.4973_4974insA) in the *MYO5A* gene [179].

Narcolepsy is a neurological disorder affecting self-control over sleep. It is distinguished by instances of drowsiness during the diurnal period, sudden episodes of sleep, disruption of sleep patterns, a shortened duration before entering rapid eye movement sleep, and occurrences of cataplexy, marked by sudden reductions or absence of skeletal muscle tone. In dogs, episodes of cataplexy are primarily induced by positive stimuli and excitement [180]. Three different variants have been identified as causal for narcolepsy in the Doberman Pinscher, Labrador Retriever, and Dachshund within the same gene (*HCRTR2*). In the Doberman Pinscher, a 226 bp SINE insertion was pinpointed as the causal variant. In the Labrador Retriever, a deletion of exon 6 was associated with narcolepsy, and, in the Dachshund, a G to A substitution at exon 1 [181,182].

Spinal dysraphisms are inherent abnormalities of the spinal cord, influenced by disruption in the intricate series of embryonic processes vital for spinal development [183]. In the Weimaraner, clinical signs were noticeable at around 2 to 4 months of age, and included paraparesis, symmetric hopping gait, hunched posture, kyphosis, and impaired proprioception in the pelvic limbs. Necropsy findings included asymmetry of the dorsal grey matter, syringomyelia, scoliosis with lateral deviation of the vertebral bodies, and other spinal cord abnormalities such as projections and asymmetry of ectopic grey matter [184]. The causal variant was a G to AA frameshift variant within exon 2 of the *NKX2-8* gene [185].

Exercise-induced collapse is a hereditary neuromuscular condition marked by exercise intolerance in otherwise healthy young adult dogs. Clinical signs are triggered by vigorous physical activity. Dogs affected by this condition typically experience a ‘wobbly’ gait within five to fifteen minutes of engaging in intense exercise. This wobbly gait then advances to nonpainful, weakened movement in the hind limbs, and the dogs lose control over them. Collapse episodes usually endure for 5 to 10 min, with most dogs fully recovering within 30 min. In rare cases, the episodes can be fatal. The variant responsible for this condition in Labrador retrievers, Chesapeake Bay Retrievers, and Curly-coated Retrievers involves a substitution from G to T in exon 6, resulting in the alteration of the amino acid codon from arginine to leucine (R256L) within a strongly conserved region of the *DNM1* gene [186].

## 3. Discussion

Most of the canine inherited neurological diseases share common signs. In addition to the clinical characteristics, it is important to consider the age of onset and the evolution of the disease. Paraclinical examination such as imaging procedures and histology may also be of aid. However, for inherited neurological conditions, the definitive diagnosis is established through genetic testing for the causal variant.

Mendelian genetics primarily concentrates on disorders that result from single genes, thereby excluding consideration of polygenic or multifactorial traits [187]. The publishing of the domestic dog genome sequence in 2005 [6] and advancements of molecular genetic techniques have made a significant contribution, as more than 200 variants leading to inherited disorders in dogs have been identified in the last 10 years [7].

The inherited neurological conditions with known causal variants in dogs that are described in this review are summarized in Table 1. It is worth noting that the majority of these diseases have an autosomal recessive pattern of inheritance. Diseases that follow such a pattern manifest when an individual inherits two recessive disease alleles. Consequently, such an individual will display the disease phenotype. In accordance with Mendel’s Law of Segregation, this implies that both parents must possess at least one allele that determines the diseases, which they can potentially transmit to their offspring [188]. 

Additionally, as per Table 1, the dog breeds that are more prone to develop such diseases are the Golden Retriever, in which six inherited neurological disorders with a known causal variant have been documented, and the Belgian Shepherd, in which five inherited neurological disorders with a known causal variant have been documented. The most documented and prevalent neurological inherited disorder is canine degenerative myelopathy (CDM). The mutant *SOD1:c.118A* allele specific to CDM was identified in more than 120 dog breeds [160]. A variant within the same SOD1 gene is also responsible for paroxysmal dyskinesia in the Markiesje dog breed [149].

**Figure 1:**
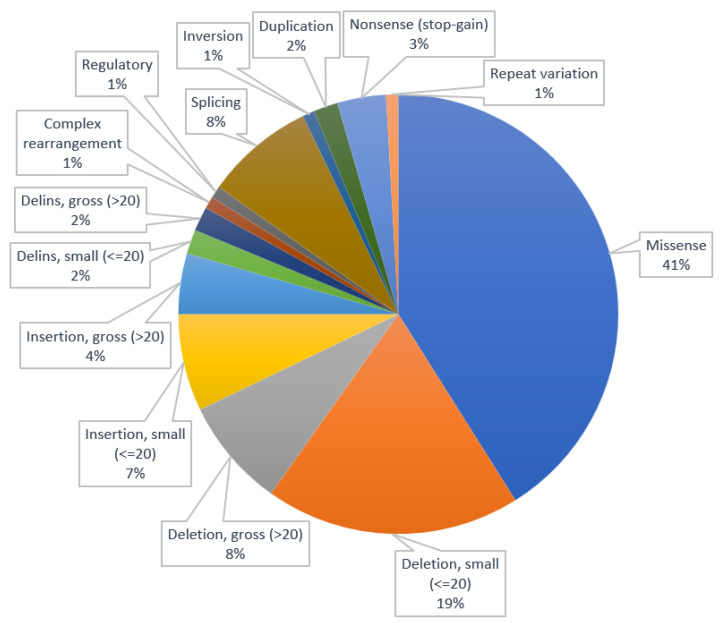
Distribution of types of variants.

The distribution of types of variants is illustrated in Figure 1. Based on the data from Table 1 and Figure 1, it is highlighted that the most frequent types of variants for canine inherited neurological disorders are the missenses (*n* = 46, 41%) and the small deletions (*n* = 21, 19%).

In order to determine the significance of the variants described in this review, we used the ABC system developed by Houge et al. [189]. This approach employs a step-by-step process, classifying genetic variants of any type. The ABC system is based on functional (A) and clinical (B) criteria, with optional inclusion of a standard comment (C) that aligns with the clinical question. Each step, A and B, employs a 1–5 grading system, with a class “zero” designation when knowledge is inadequate. Functional grading (A) is concerned with the biological consequences of the variant, and includes stages from normal function (1) to proven functional effect (5). Clinical grading (B) focuses on the genotype–phenotype relationship, categorizing variants as “right type of gene” (1), risk factor (2), or pathogenic (3–5, depending on penetrance). Combining the A and B grading results in the generation of A–F classes, which are correlated with standard comments that reflect national or laboratory policies [189]. Instead of using the standard comments, we replaced them with our own comments, as per the following: “variant of unknown significance” for the class F variants, “likely pathogenic variant” for class E and D variants, and “pathogenic variant” for classes C, B, and A variants. The detailed classification is presented in Table S1. Based on our assessment, 16 of the variants were classified as pathogenic variants and the remaining 96 were classified as likely pathogenic variants.

A recent research study screened the largest cohort of canines to date, comprising 1,054,293 representative dogs, to investigate the prevalence and distribution of a total of 250 genetic-disease-associated variants within the general population [113]. The study included 811,628 mixed breed dogs and 242,665 purebreds. There was a noted connection, although with a small correlation coefficient, between a widespread reduction in genetic diversity and an increased likelihood of homozygosity for multiple autosomal-recessive-disease-related variants in dogs. This finding provides a concrete, quantifiable example of how elevated genomic homozygosity, which can occur through close interbreeding within a restricted gene pool, may potentially lead to the occurrence of inbreeding depression as a result of the accumulation of harmful recessive alleles. While it is theoretically possible for disease-associated variants to persist in mixed breed populations through random breeding, with their frequencies fluctuating over time due to random genetic drift, it is important to note that matings between closely related individuals, such as those in puppy mills, are known to occur and can play a role in the maintenance of these variants. Additionally, the study revealed that 57% of the dogs tested carried at least one copy of one of the studied Mendelian-disease-associated variants. It is worth highlighting that the majority of the specific variants included in this study have a frequency of less than 1% in the population [190]. 

Functional testing of genetic variations is vital for progressing the comprehension of the genetic origins of illnesses and directing customized treatment methods. Whole-exome sequencing (WES), whole-genome sequencing (WGS), GWAS, and RNA sequencing have progressively become common methodologies for diagnosing Mendelian diseases. 

Despite their effectiveness, the existing diagnostic rate for genomic analyses encompassing various rare diseases is around 25 to 50%. There are several potential outcomes of employing a WES or WGS strategy: identification of a known disease-causing variant in a disease gene associated with a clinical phenotype that aligns with the patient’s condition under investigation, identification of an unknown variant in a known disease gene with a corresponding clinical phenotype, identification of a known variant in a known disease gene with a dissimilar phenotype, identification of an unknown variant in a known disease gene with a dissimilar phenotype, identification of an unknown variant in a gene not previously linked to any disease, and failure to detect any genetic variant that can account for the patient’s phenotype [191]. 

The GWAS has emerged over the last 15 years as a primary method for pinpointing the genetic variations linked to complex traits. These studies have linked thousands of variants to hundreds of phenotypes, significantly advancing the comprehension of the genetic framework underpinning complex diseases [192,193]. 

The transcriptome represents the comprehensive collection of transcripts within a cell, including their quantities, during a specific developmental stage or physiological state. Comprehending the transcriptome is vital for interpreting the functional components of the genome, unraveling the molecular constituents of cells and tissues, and gaining insights into both developmental processes and disease mechanisms. The primary objectives of transcriptomics are as follows: to create a comprehensive inventory of all types of transcripts, encompassing mRNAs, non-coding RNAs, and small RNAs; to ascertain the transcriptional structure of genes, including their start sites, 5′ and 3′ ends, splicing patterns, and other post-transcriptional modifications; and to quantify the dynamic changes in the expression levels of each transcript during development and across various conditions [194].

## 4. Conclusions

This review aims to summarize the current knowledge on the canine nervous system phenes and their genetic causal variant. The majority of these diseases have an autosomal recessive pattern of inheritance. Golden Retrievers and Belgian Shepherds were the breeds with the biggest number of documented neurological diseases with known causal variants.

Genetic testing can play a vital role in effectively managing and ultimately eradicating inherited diseases. Recessive diseases pose a particular challenge for dog breeders because the parents are often asymptomatic carriers in the population who may only be identified retrospectively, typically after they have already given birth to affected puppies or in some cases after one of their parents has been diagnosed with the disease. This challenge is further complicated when dealing with late-onset disorders, where affected dogs may unknowingly be bred before receiving a diagnosis. This issue applies to both dominant and recessive diseases.

Given that the majority of variants have low frequencies, it can be challenging for any individual veterinary clinician to identify and stay proficient in diagnosing the wide range of characterized inherited disorders. This underscores the importance of ongoing medical training and the presence of veterinary specialists with specific expertise in genetic counseling. It also emphasizes the importance of comprehensive diagnostic screening technologies.

## Figures and Tables

**Table 1:** Inherited neurological conditions with known causal variants in dogs [7], adapted by the authors.

Phene	Mode of Inheritance	Involved Chromosome	Concerned Gene and Its Description	Type of Variant	Affected Dog Breeds
Acral mutilation syndrome	Autosomal recessive	4	*GDNF*—glial cell derived neurotrophic factor	Regulatory	English Pointer, English Springer Spaniel, French Spaniel, German Shorthaired Pointer [114]
Medium-chain acyl-CoA dehydrogenase deficiency	Autosomal recessive	6	*ACADM*—acyl-CoA dehydrogenase, C-4 to C-12 straight chain	Delins, gross (>20)	Cavalier King Charles Spaniel [177]
Alexander disease	Autosomal dominant	9	*GFAP*—glial fibrillary acidic protein	Missense	Labrador Retriever [171]
Cerebellar ataxia	Autosomal recessive	5	*ATP1B2*—ATPase, Na^+^/K^+^ transporting, beta 2 polypeptide	Insertion, gross (>20)	Belgian Shepherd [17]
Cerebellar ataxia	Autosomal recessive	4	*RAB24*—RAB24, member RAS oncogene family	Missense	Gordon Setter, Old English Sheepdog [18]
Cerebellar ataxia	Autosomal recessive	3	*KCNIP4*—Kv channel interacting protein 4	Missense	Norwegian Buhund [19]
Cerebellar ataxia	Autosomal recessive	38	*KCNJ10*—potassium channel, inwardly rectifying subfamily J, member 10	Missense	Malinois, Jack Russell Terrier Parson, Russell Terrier [13,14]
Cerebellar ataxia	Autosomal recessive	1	*GRM1*—glutamate receptor, metabotropic 1	Insertion, gross (>20)	Coton de Tulear [21]
Cerebellar ataxia	Autosomal recessive	8	*SEL1L*—sel-1 suppressor of lin-12-like (*C. elegans*)	Missense	Finnish Hound [23]
Cerebellar ataxia	Autosomal recessive	8	*RALGAPA1*—Ral GTPase activating protein, alpha subunit 1 (catalytic)	Deletion, gross (>20)	Belgian Shepherd [15]
Cerebellar ataxia	Autosomal recessive	4	*SEPP1*—selenoprotein P, plasma, 1	Deletion, gross (>20)	Belgian Shepherd [16]
Ataxia	Autosomal recessive	12	*HACE1*—HECT domain and ankyrin repeat containing E3 ubiquitin protein ligase 1	Deletion, small (≤20)	Norwegian Elkhound [24]
Spinocerebellar ataxia	Autosomal recessive	18	*CAPN1*—calpain 1, (mu/I) large subunit	Missense	Parson Russell Terrier [25]
Spinocerebellar ataxia	Autosomal recessive	20	*ITPR1*—inositol 1,4,5-trisphosphate receptor, type 1	Complex rearrangement	Italian Spinone [26]
Spinocerebellar ataxia	Autosomal recessive	27	*SCN8A*—sodium channel, voltage-gated, type VIII alpha subunit	Missense	Alpine Dachsbracke [27]
Spinocerebellar ataxia	Autosomal recessive	30	*SLC12A6*—solute carrier family 12 (potassium/chloride transporter), member 6	Delins, small (≤20)	Belgian Shepherd [28]
Spinocerebellar ataxia	Autosomal recessive	18	*SPTBN2*—spectrin, beta, non-erythrocytic 2	Deletion, small (≤20)	Beagle [29]
Cerebellar abiotrophy	Autosomal recessive	9	*VMP1*—vacuole membrane protein 1	Missense	Australian Working Kelpie [30]
Cerebellar cortical degeneration	Unknown	12	*SNX14*—sorting nexin 14	Splicing	Vizsla [31]
Cerebellar degeneration–myositis complex	Unknown	36	*SLC25A12*—solute carrier family 25 (aspartate/glutamate carrier), member 12	Missense	Nova Scotia Duck Tolling Retriever [32]
Cerebellar hypoplasia	Autosomal recessive	1	*VLDLR*—very low density lipoprotein receptor	Deletion, small (≤20)	Eurasier [33]
Degenerative myelopathy	Autosomal recessive	31	*SOD1*—superoxide dismutase 1	Missense	Multiple breeds [160]
Dilute coat color with neurological defects	Autosomal recessive	30	*MYO5A*—myosin VA	Insertion, small (≤20)	Miniature Dachshund [179]
Paroxysmal dyskinesia	Autosomal recessive	1	*PIGN*—phosphatidylinositol glycan anchor biosynthesis, class N	Missense	Soft-Coated Wheaten Terrier [148]
Paroxysmal dyskinesia	Autosomal recessive	31	*SOD1*—superoxide dismutase 1	Delins, small (≤20)	Markiesje [149]
Paroxysmal dystonia–ataxia syndrome	Autosomal recessive	7	*TNR*—tenascin R	Insertion, small (≤20)	Weimaraner [153]
Episodic falling	Autosomal recessive	7	*BCAN*—brevican	Deletion, gross (>20)	Cavalier King Charles Spaniel [154]
Exercise-induced collapse	Autosomal recessive	9	*DNM1*—dynamin 1	Missense	Chesapeake Bay Retriever, Curly-Coated Retriever, Labrador Retriever [186]
Hyperekplexia	Autosomal recessive	21	*SLC6A5*—solute carrier family 6 member 5	Deletion, gross (>20)	Irish Wolfhound [151]
Hyperekplexia	Autosomal recessive	4	*GLRA1*—glycine receptor alpha 1	Deletion, gross (>20)	Miniature Australian Shepherd [152]
Hypomyelination of the central nervous system	Autosomal recessive	15	*FNIP2*—folliculin interacting protein 2	Deletion, small (≤20)	Weimaraner [164]
L-2-hydroxyglutarate dehydrogenase	Autosomal recessive	8	*L2HGDH*—L-2-hydroxyglutarate dehydrogenase	Missense	Yorkshire Terrier [172]
Laryngeal paralysis and polyneuropathy	Autosomal recessive	9	*CNTNAP1*—contactin associated protein 1	Missense	Labrador Retriever, Leonberger, Pyrenean Shepherd, Saint Bernard [123]
Leukodystrophy	Mitochondrial	N/A	*CYTB*—cytochrome b	Missense	Australian Cattle Dog, Shetland Sheepdog [168]
Leukodystrophy	Autosomal recessive	9	*TSEN54*—TSEN54 tRNA splicing endonuclease subunit	Missense	Standard Schnauzer [169]
Leukoencephalomyelopathy	Autosomal recessive	18	*NAPEPLD*—N-acyl phosphatidylethanolamine phospholipase D	Insertion, small (≤20)	Great Dane, Rottweiler [170]
Lissencephaly and cerebellar hypoplasia	Probably autosomal recessive	18	*RELN*—reelin	Deletion, small (≤20)	White Swiss Shepherd [178]
Multiple system degeneration	Autosomal recessive	1	*SERAC1*—serine active site containing 1	Splicing	Chinese Crested [12]
Congenital myasthenic syndrome	Autosomal recessive	28	*CHAT*—choline O-acetyltransferase	Missense	Old Danish Pointer [127]
Congenital myasthenic syndrome	Autosomal recessive	5	*CHRNE*—cholinergic receptor, nicotinic, epsilon (muscle)	Insertion, small (≤20)	Heideterrier, Jack Russell Terrier [125,126]
Generalized myoclonic epilepsy with photosensitivity	Autosomal recessive	20	*DIRAS1—*DIRAS family, GTP-binding RAS-like 1	Deletion, small (≤20)	Rhodesian Ridgeback [130]
Benign familial juvenile epilepsy	Autosomal dominant with incomplete penetrance	3	*LGI2—*leucine rich repeat LGI family member 2	Nonsense (stop-gain)	Lagotto Romagnolo [132]
Myoclonus epilepsy of Lafora	Autosomal recessive	35	*NHLRC1—*NHL repeat containing E3 ubiquitin protein ligase 1	Repeat variation	Beagle, Chihuahua, Miniature Wirehaired Dachshund, Newfoundland, Pembroke Welsh Corgi [135,136,137,138]
Epilepsy with mitochondrial dysfunction and neurodegeneration	Autosomal recessive	2	*PITRM1—*pitrilysin metallopeptidase 1	Deletion, small (≤20)	Parson Russell Terrier [139]
Deafness	Unknown	37	*KLF7—*Kruppel-like transcription factor 7	Missense	Australian Stumpy Tail Cattle Dogs [141]
Deafness	Autosomal recessive	4	*CDH23—*cadherin-related 23	Missense	Beauceron [142]
Deafness	Autosomal recessive	18	*EPS8L2—*EPS8-like 2	Deletion, small (≤20)	Rhodesian Ridgeback [143]
Deafness	Autosomal recessive	7	*LOXHD1—*lipoxygenase homology PLAT domains 1	Missense	Rottweiler [144]
Bilateral deafness and vestibular dysfunction	Autosomal recessive	21	*MYO7A—*myosin VIIA	Missense	Doberman Pinscher [146]
Unilateral deafness and vestibular dysfunction	Autosomal recessive	15	*PTPRQ—*protein tyrosine phosphatase receptor type Q	Insertion, small (≤20)	Doberman Pinscher [147]
Myeloencephalopathy degenerative progressive	Autosomal recessive	18	*PNPLA8*—patatin-like phospholipase domain containing 8	Duplication	Australian Shepherd [34]
Subacute necrotising encephalopathy	Autosomal recessive	25	*SLC19A3*—solute carrier family 19 (thiamine transporter), member 3	Delins, gross (>20)	Alaskan Husky [156]
Necrotising myelopathy	Autosomal recessive	14	*IBA57*—homolog, iron-sulfur cluster assembly	Missense	Dutch Kooiker [158]
Neonatal encephalopathy with seizures	Autosomal recessive	36	*ATF2*—activating transcription factor 2	Missense	Standard Poodle [155]
Neuroaxonal dystrophy	Autosomal recessive	2	*MFN2*—mitofusin 2	Deletion, small (≤20)	Schnauzer–Beagle cross [107]
Neuroaxonal dystrophy	Autosomal recessive	10	*PLA2G6*—phospholipase A2, group VI (cytosolic, calcium-independent)	Missense	Papillon [104]
Neuroaxonal dystrophy	Autosomal recessive	8	*TECPR2*—tectonin beta-propeller repeat containing 2	Missense	Spanish Water Dog [106]
Neuroaxonal dystrophy	Autosomal recessive	5	*VPS11*—vacuolar protein sorting 11 homolog	Missense	Rottweiler [105]
Lysosomal storage disease	Autosomal incomplete dominant	9	CNP—2′,3′-cyclic-nucleotide 3′-phosphodiesterase	Deletion, small (≤20)	Dalmatian [59]
Lysosomal storage disease	Autosomal recessive	9	ARSG—arylsulfatase G	Missense	American Staffordshire Terrier [78]
Mucopolysaccharidosis I	Autosomal recessive	3	IDUA—alpha-L-iduronidase	Splicing	Plott Hound [62]
				Insertion, small (≤20)	Boston Terrier [63]
				Deletion, gross (>20)	Golden Retriever [64]
Mucopolysaccharidosis IIIA	Autosomal recessive	9	*SGSH—*N-sulfoglucosamine sulfohydrolase	Deletion, small (≤20)	Dachshund [67]
				Insertion, small (≤20)	New Zealand Huntaway Dog [65]
Mucopolysaccharidosis IIIB	Autosomal recessive	9	*NAGLU—*N-acetyl-alpha-glucosaminidase	Insertion, gross (>20)	Schipperke [70]
Mucopolysaccharidosis VI	Autosomal recessive	3	*ARSB—*arylsulfatase B	Deletion, gross (>20)	Miniature Poodle [72]
				Nonsense (stop-gain)	Great Dane [73]
				Deletion, gross (>20)	Miniature Schnauzer [74]
				Missense	Miniature Pinscher [74]
Mucopolysaccharidosis VII	Autosomal recessive	6	*GUSB—*glucuronidase beta	Missense	German Shepherd [76]
				Missense	Brazilian Terrier [77]
Alpha-mannosidosis	Probably autosomal recessive	20	*MAN2B1—*mannosidase alpha class 2B member 1	Missense	Doberman Pinscher [81]
Beta-mannosidosis	Autosomal recessive	32	*MANBA—*mannosidase beta	Missense	German Shepherd [83]
				Duplication	Mixed dog breed [84]
GM1 gangliosidosis	Autosomal recessive	23	*GLB1—*galactosidase beta 1	Missense	Portuguese Water Dog [88]
				Deletion, small (≤20)	Miniature Shiba, Shiba Inu [89]
				Insertion, small (≤20)	Alaskan Husky [91]
GM2 gangliosidoses	Autosomal recessive	30	*HEXA—*hexosaminidase subunit alpha	Missense	Japanese Chin [92]
GM2 gangliosidosis type II	Autosomal recessive	2	*HEXB—*hexosaminidase subunit beta	Deletion, small (≤20)	Toy Poodle [93]
				Deletion, small (≤20)	Shiba Inu [94]
Glycogen storage disease II	Autosomal recessive	9	*GAA—*alpha glucosidase	Nonsense (stop-gain)	Finnish Lapphund, Swedish Lapphund [96]
Alpha fucosidosis	Autosomal recessive	2	*FUCA1—*alpha-L-fucosidase 1	Deletion, small (≤20)	English Springer Spaniel [98]
Krabbe disease	Autosomal recessive	8	*GALC—*galactosylceramidase	Missense	Cairn Terrier, West Highland White Terrier [100]
				Insertion, gross (>20)	Irish Setter [101]
				Missense	Mixed dog breed [102]
Neurodegenerative vacuolar storage disease	Autosomal recessive	20	*ATG4D*—autophagy-related 4D, cysteine peptidase	Missense	Lagotto Romagnolo [35]
Neuronal ceroid lipofuscinosis, type 1	Autosomal recessive	15	*PPT1*—palmitoyl-protein thioesterase 1	Splicing	Italian Cane Corso [41]
Neuronal ceroid lipofuscinosis, type 2	Autosomal recessive	21	*TPP1*—tripeptidyl peptidase I	Deletion, small (≤20)	Dachshund [42]
Neuronal ceroid lipofuscinosis, type 5	Autosomal recessive	22	*CLN5*—ceroid-lipofuscinosis, neuronal 5	Deletion, small (≤20)	Golden Retriever [45]
Neuronal ceroid lipofuscinosis, type 6	Autosomal recessive	30	*CLN6*—ceroid-lipofuscinosis, neuronal 6, late infantile, variant	Missense	Australian Shepherd [46]
Neuronal ceroid lipofuscinosis, type 7	Autosomal recessive	19	*MFSD8*—major facilitator superfamily domain containing 8	Deletion, small (≤20)	Chihuahua, Chinese Crested [47]
Neuronal ceroid lipofuscinosis, type 8	Autosomal recessive	37	*CLN8*—ceroid-lipofuscinosis, neuronal 8 (epilepsy, progressive with mental retardation)	Deletion, gross (>20)	Alpine Dachsbracke [52]
Neuronal ceroid lipofuscinosis, type 10	Autosomal recessive	18	*CTSD*—cathepsin D	Missense	American Bulldog [56]
Neuronal ceroid lipofuscinosis, type 12	Autosomal recessive	2	*ATP13A2*—ATPase type 13A2	Splicing	Tibetan Terrier [57]
				Missense	Australian Cattle Dog [58]
Hereditary sensory and autonomic neuropathy	Autosomal recessive	26	*SCN9A*—sodium channel, voltage-gated, type IX, alpha subunit	Missense	Mixed dog breed [110]
Sensory ataxic neuropathy	Mitochondrial	N/A	*MTTY*—mitochondrially encoded tRNA tyrosine	Deletion, small (≤20)	Golden Retriever [111]
Sensory neuropathy	Autosomal recessive	4	*FAM134B*—family with sequence similarity 134, member B	Inversion	Border Collie [113]
Polyneuropathy	Probably autosomal recessive	16	*ARHGEF10*—Rho guanine nucleotide exchange factor (GEF) 10	Deletion, small (≤20)	Leonberger, Saint Bernard [115]
Polyneuropathy	Autosomal incomplete dominant	15	*GJA9*—gap junction protein, alpha 9, 59kDa	Deletion, small (≤20)	Leonberger [116]
Hypomyelinating polyneuropathy	Probably autosomal dominant	38	*MPZ*—myelin protein zero	Missense	Golden Retriever [162]
Hypomyelinating polyneuropathy	Probably autosomal recessive	21	*MTMR2*—myotubularin-related protein 2	Splicing	Golden Retriever [162]
Hypomyelinating polyneuropathy	Probably autosomal recessive	4	*SH3TC2*—SH3 domain and tetratricopeptide repeats 2	Nonsense (stop-gain)	Golden Retriever [162]
Polyneuropathy	Autosomal recessive	13	*NDRG1*—N-myc downstream regulated 1	Missense	Alaskan Malamute [119]
Polyneuropathy with ocular abnormalities and neuronal vacuolation	Autosomal recessive	19	*RAB3GAP1*—RAB3 GTPase activating protein subunit 1 (catalytic)	Insertion, gross (>20)	Alaskan Husky [122]
Polyneuropathy	Autosomal recessive	21	*SBF2*—SET binding factor 2	Splicing	Miniature Schnauzer [117]
Succinic semialdehyde dehydrogenase deficiency	Autosomal recessive	25	*ALDH5A1*—aldehyde dehydrogenase 5 family, member A1	Missense	Saluki [175]
Narcolepsy	Autosomal recessive	12	*HCRTR2—*hypocretin receptor 2	Splicing	Doberman Pinscher [182]
				Splicing	Labrador Retriever [182]
				Missense	Dachshund [181]
X-linked tremor	X-linked recessive	X	*PLP1*—proteolipid protein 1	Missense	Springer Spaniel [166]

## Data Availability

Not applicable.

## Supplementary Materials

The following supporting information can be downloaded at: https://www.mdpi.com/article/10.3390/ani13223568/s1, Table S1: Modified ABC variant classification.
